# Prenatal ethanol exposure increases osteoarthritis susceptibility in female rat offspring by programming a low-functioning IGF-1 signaling pathway

**DOI:** 10.1038/srep14711

**Published:** 2015-10-05

**Authors:** Qubo Ni, Yang Tan, Xianrong Zhang, Hanwen Luo, Yu Deng, Jacques Magdalou, Liaobin Chen, Hui Wang

**Affiliations:** 1 Department of Pharmacology, Basic Medical School of Wuhan University, Wuhan 430071, China; 2 Department of Orthopedic Surgery, Zhongnan Hospital of Wuhan University, Wuhan 430071, China; 3 Hubei Provincial Key Laboratory of Developmentally Originated Disease, Wuhan 430071, China; 4 Université de Lorraine, Ingénierie Moléculaire, Physiopathologie Articulaire (IMoPA), UMR 7365 CNRS, Biopôle, F-54505 Vandœuvre-lès-Nancy, France

## Abstract

Epidemiological evidence indicates that osteoarthritis (OA) and prenatal ethanol exposure (PEE) are both associated with low birth weight but possible causal interrelationships have not been investigated. To investigate the effects of PEE on the susceptibility to OA in adult rats that experienced intrauterine growth retardation (IUGR), and to explore potential intrauterine mechanisms, we established the rat model of IUGR by PEE and dexamethasone, and the female fetus and 24-week-old adult offspring subjected to strenuous running for 6 weeks were sacrificed. Knee joints were collected from fetuses and adult offspring for histochemistry, immunohistochemistry and qPCR assays. Histological analyses and the Mankin score revealed increased cartilage destruction and accelerated OA progression in adult offspring from the PEE group compared to the control group. Immunohistochemistry showed reduced expression of insulin-like growth factor-1 (IGF-1) signaling pathway components. Furthermore, fetuses in the PEE group experienced IUGR but exhibited a higher postnatal growth rate. The expression of many IGF-1 signaling components was downregulated, which coincided with reduced amounts of type II collagen in the epiphyseal cartilage of fetuses in the PEE group. These results suggest that PEE enhances the susceptibility to OA in female adult rat offspring by down-regulating IGF-1 signaling and retarding articular cartilage development.

Osteoarthritis (OA) is the most common cause of joint pain and mobility disability during aging and is characterized by the major pathological feature of articular cartilage degeneration^1^. OA affects the quality of life of patients and places a great economic burden on families and society. In 2010, nearly 33.6% of adults aged 65 years or older in developed countries suffered from OA. Moreover, the incidence rate increases yearly, and the current age of onset is younger than that in previous decades^2^. Despite the high incidence of OA, its etiology and pathogenesis are unclear. Epidemiological data showed that hand OA and lumbar spine OA were significantly associated with low birth weight^3^,^4^. Cartilage dysplasia can induce OA because it alters joint geometry, mechanical loading and cartilage matrix composition^5^. These reports indicate that changes in cartilage during the intrauterine development period increase the susceptibility to OA.

Intrauterine growth retardation (IUGR) based on birth weight is an important index for evaluating birth status. IUGR is defined as a birth weight and/or length less than the 10th percentile or less than two standard deviations below the mean at the same gestational age^6^,^7^. The incidence of IUGR is as high as 24% in developing countries, approximately 30 million newborns suffer from IUGR each year; Asia is the most impacted region, accounting for nearly 75% of all cases^8^. Studies have shown that fetal weight is positively correlated with the serum level of insulin-like growth factor-1 (IGF-1)^9^. IGF-1 deficiency leads to severe intrauterine and postnatal growth retardation, perinatal mortality and developmental defects^10^. IGF-1 is also a key factor in cartilage anabolism and the maintenance of the cartilage phenotype; it enhances chondrocyte proliferation and differentiation and promotes the synthesis of extracellular matrix^11^. Hence, deficiencies in IGF-1 and its downstream pathways, such as the PI3K/AKT pathway, may lead to articular cartilage dysplasia.

Ethanol is one of the most definite and dangerous causes of IUGR^12^. Studies indicate that children with prenatal alcohol exposure show growth retardation in the womb, infancy, and adulthood^13^. Ethanol abuse decreases IGF-1 bioavailability and alters IGF-1 signaling in cardiomyocytes^14^,^15^. Our previous studies found that prenatal ethanol exposure (PEE) results in fetal exposure to high levels of maternal glucocorticoids (GC), reduces fetal blood and liver IGF-1 levels and increases the incidence of IUGR^12^,^16^. IUGR adult offspring are more likely to suffer from metabolic syndrome (MS) and metabolic diseases, such as non-alcoholic fatty liver disease^16^,^17^. One of the possible underlying mechanisms involves the intrauterine programming of low IGF-1 signaling that is induced by over-exposure to maternal GC. Taken together, these previous findings indicate that PEE-induced excessive fetal exposure to maternal GC can downregulate blood levels of IGF-1 and local cartilage IGF-1 signaling, which results in articular cartilage dysplasia, reduces the quality of the articular cartilage after birth and increases the susceptibility to OA in adult offspring.

The present study aimed to confirm the effect of PEE on the susceptibility to OA in IUGR adult rat offspring. We used prenatal dexamethasone (DEX) exposure (PDE) as the positive control for increased fetal exposure to blood GC induced by PEE to examine the expression of the articular cartilage IGF-1 pathway and thus elucidate the underlying mechanism of the fetal origin of PEE-induced OA. This study may increase the understanding of the etiopathogenesis of adult OA and provide evidence for the development of novel strategies for the early prevention and treatment of OA of fetal origin.

## Results

### From postnatal stage to adulthood

#### Body weight changes after birth

**Figure 1:**
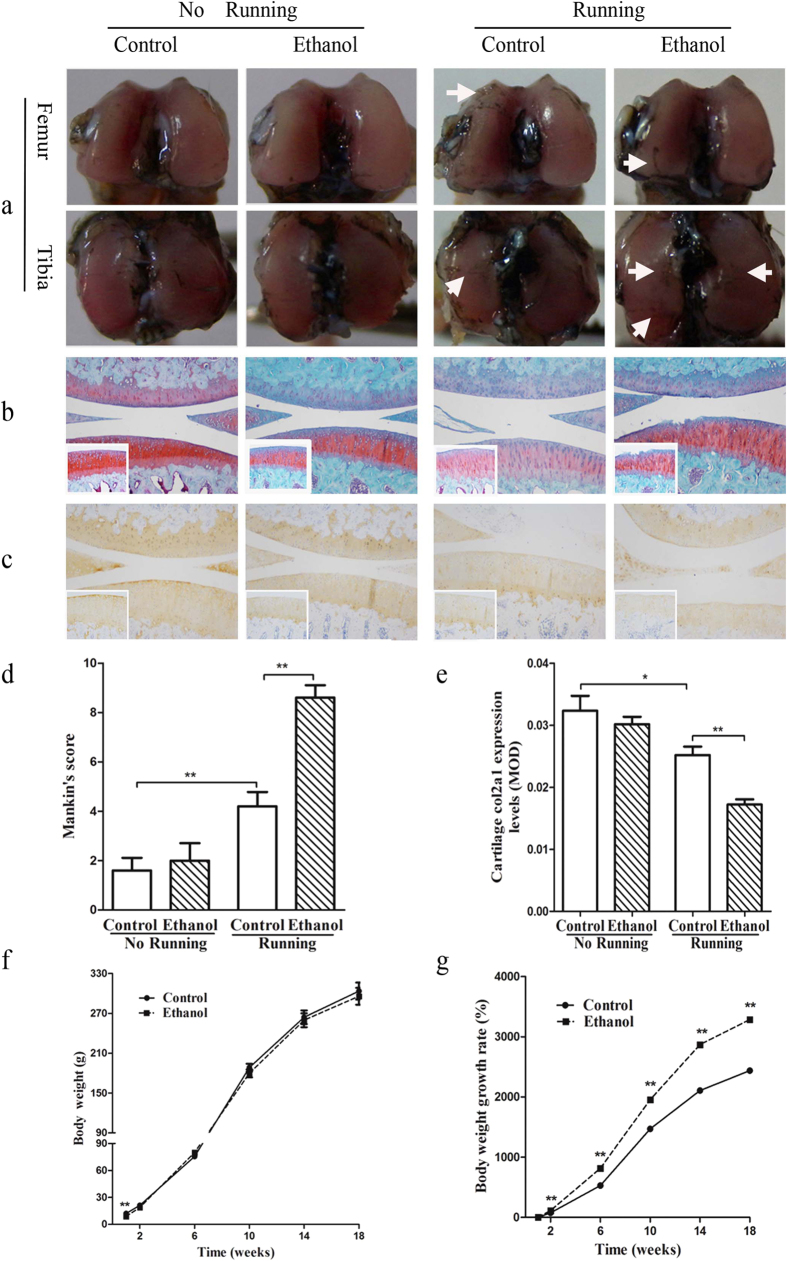
Effects of prenatal ethanol exposure on postnatal body weight and knee joint cartilage histology and function after strenuous running in adult female rat offspring. (**a**) India ink staining (femoral condyle and tibial plateau, 40×); (**b**) Safranin O/fast green staining (knee joint, 100×; tibial cartilage, 200×); (**c**) Immunohistochemical analysis of collagen type II alpha 1 (Col2A1) expression (knee joint, 100×; tibial cartilage, 200×); (**d**) Mankin score; (**e**) Mean optical density (MOD) of Col2a1 expression. The results suggest that osteoarthritis susceptibility was increased in the adult offspring with intrauterine growth retardation induced by prenatal ethanol exposure (n = 5 offspring from 8 pregnant rats). (**f**) Postnatal body weight; (**g**) Rates of weight gain (n = 16 offspring from 8 pregnant rats). Body weight and the corresponding growth rate were assessed by two-way ANOVA with repeated measurements. Mean ± S.E.M.; ^*^P < 0.05, ^**^P < 0.01 vs. corresponding controls.

Compared to the control group, the offspring in the PEE group had a significantly lower body weight at postnatal week 1 (PW1) (*P* < 0.01, Fig. 1f); thereafter, there was no significant difference in the longitudinal changes in body weight between these two groups. However, the corresponding growth rates of the PEE group were significantly higher (Fig. 1g; *P* < 0.01, two-way ANOVA with repeated measurements). These results suggest that PEE leads to low birth weight, and these low-birth-weight fetuses can undergo catch-up growth^18^ after birth.

### Histological changes in the cartilage after treadmill running

As shown in Fig. 1, the running rats in the control group showed a rougher articular surface as indicated by increased India ink staining, reduced Safranin O staining, and an increased Mankin score (*P* < 0.01; Fig. 1d). In addition, reduced immunohistochemical staining of collagen type II alpha 1 (Col2a1) was observed, and the corresponding mean optical density (MOD) was lower (*P* < 0.05; Fig. 1c,e) in strenuously running rats. These results confirmed the successful creation of the strenuous running-induced knee joint OA model.

The articular cartilage histology results were as follows.

(1) In the non-running PEE group, the overall cartilage organization was normal, the cartilage surfaces were smooth without ink staining, and empty chondrocyte lacunae and fibrillated areas were not present, similar to the non-running control group (Fig. 1a,b). However, after treadmill running, the cartilage surface, especially the tibial plateau surface, appeared to have much more wear and tear in the PEE group than in the control group (Fig. 1a).

(2) In the non-running rats, the surfaces and deep zone of the cartilage were normal, and there was only a slight reduction in Safranin O staining in the PEE group. After treadmill running, varying degrees of erosion and irregularity in the articular cartilage were observed in the PEE group, including the appearance of clefts, reduced cartilage thickness, lightly stained matrix, a reduced number of chondrocytes, and blurred tidemarks (Fig. 1b).

(3) In the non-running rats, the Mankin score of the PEE group showed an increasing trend compared to that of the control group (*P* > 0.05). However, after treadmill running to induce OA, the cartilage pathological Mankin score was significantly higher in the PEE group compared to the control group (*P* < 0.01; Fig. 1d).

(4) In the non-running rats, there was no obvious difference in Col2a1 staining in the PEE group, and the corresponding MOD showed a decreasing trend. However, after treadmill running, the MOD of Col2a1 staining was significantly decreased to 68.3% of that observed in the control rats (Fig. 1c,e). These results suggest that OA susceptibility was increased in the adult offspring with PEE-induced IUGR.

#### IGF-1 pathway-related protein expression changes in adult articular cartilage

**Figure 2:**
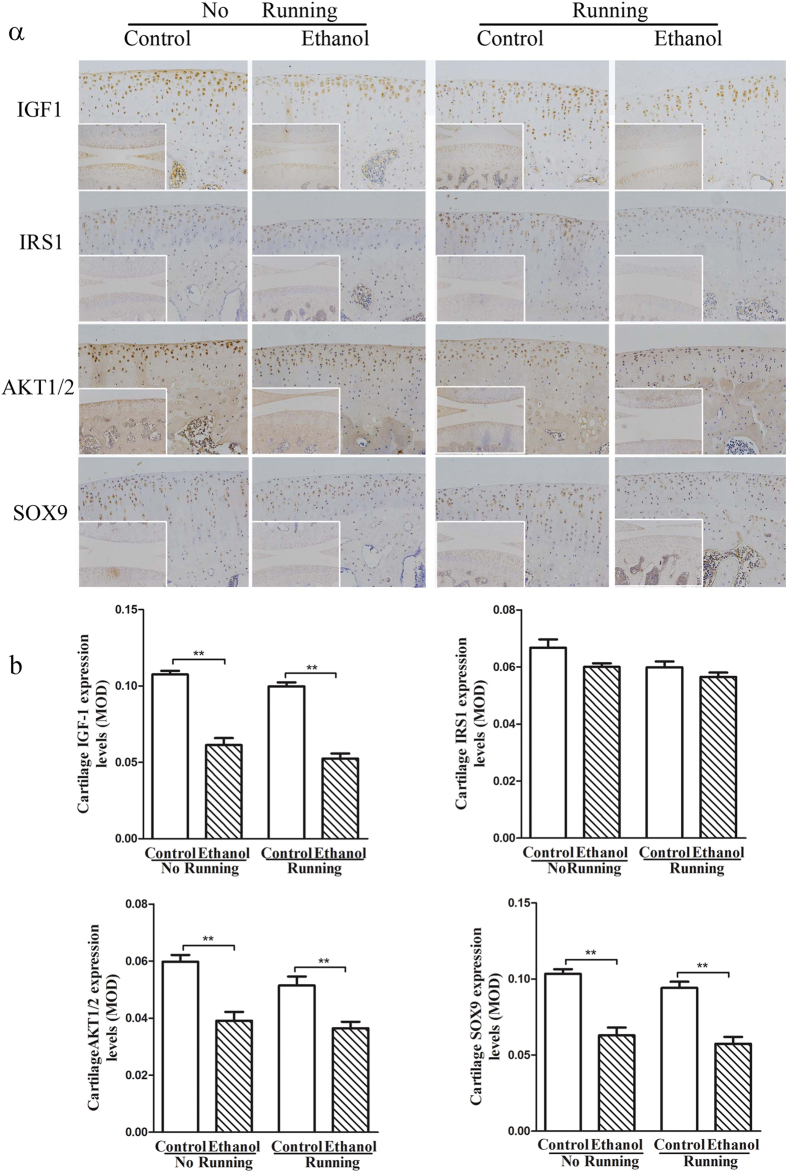
Effects of prenatal ethanol exposure and adult strenuous running on the protein expression of insulin-like growth factor-1 (IGF-1) pathway components (insulin receptor substrate 1 (IRS1), serine–threonine protein kinase 1/2 (AKT1/2) and SRY (sex determining region Y)-box 9 (SOX9)) in the articular cartilage of adult female offspring. (**a**) Representative images from each group (knee joint, 100×; tibial cartilage, 200×); (**b**) Mean optical density (MOD) of each group. Mean ± S.E.M.; n = 5 offspring from 8 pregnant rats. ^**^P < 0.01 vs. corresponding controls.

Immunohistochemistry showed that the expression of IGF-1, serine–threonine protein kinase 1/2 (AKT1/2) and SRY (sex determining region Y)-box 9 (SOX9) was significantly decreased (*P* < 0.01) in the articular cartilage of the adult rats in PEE group compared with that in the non-running control rats (Fig. 2a). Quantification of the staining revealed that the expression of these 3 proteins was reduced to 57%, 65%, and 61%, respectively (*P* < 0.01, Fig. 2b). The protein expression of IGF-1, AKT and SOX9 in the articular cartilage of adult rats in the PEE group was significantly decreased compared to that in running rats in the control group (*P* < 0.01). These results indicate that PEE reduced the protein expression of the IGF-1 pathway in adult articular cartilage.

### Intrauterine period

#### Fetal body weight and blood metabolic phenotype changes

**Figure 3:**
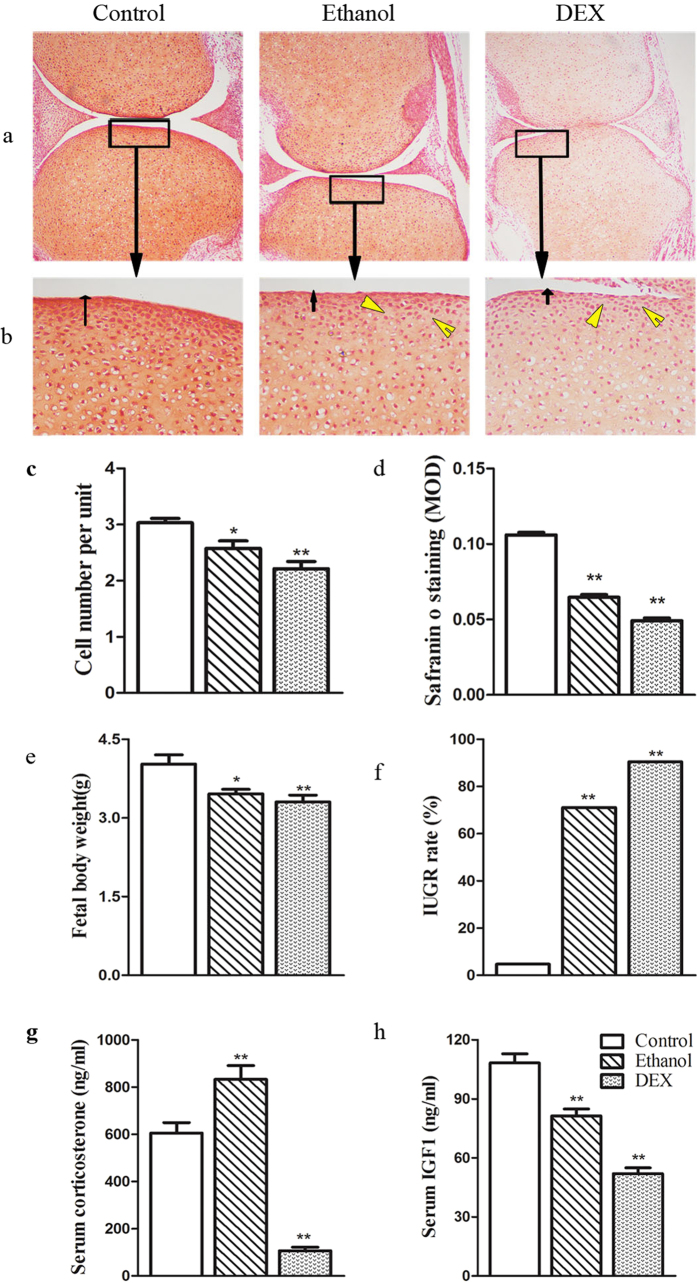
Effect of prenatal ethanol or dexamethasone (DEX) exposure on body weight, serum corticosterone and insulin-like growth factor 1 (IGF-1) levels and histological changes in articular cartilage in female fetal rats. (**a**) Safranin O/fast green staining of the knee joint (100×); (**b**) Safranin O/fast green staining of the tibial articulating superficial zone (400×). Ethanol and DEX exposure resulted in a thin layer of articular cartilage (black arrowheads) and histological gaps (yellow arrows); (**c**) Cell count within the knee joint cartilage area; (**d**) Mean optical density (MOD) of the proteoglycan content in the tibial articulating superficial zone; (**e**) Body weight; (**f**) Intrauterine growth retardation (IUGR) rates; (**g**) Serum corticosterone concentrations; (**h**) Serum IGF-1 concentrations (n = 5 litters from 8 pregnant rats). Mean ± S.E.M.; n = 5 offspring from 8 pregnant rats. ^*^P < 0.05, ^**^P < 0.01 vs. control.

The fetal body weights in the PEE and PDE groups were reduced to 85.9% and 82.1% compared to the control group (*P* < 0.05, *P* < 0.01; Fig. 3e), and the IUGR rates were increased from 4.7% (control) to 71.1% and 90.4%, respectively (*P* < 0.01; Fig. 3f). The serum corticosterone (CORT) levels were significantly higher in the PEE group compared to the control group (*P* < 0.01), but the CORT levels were significantly lower in the PDE group compared to the control group (*P* < 0.01; Fig. 3g). Moreover, the serum levels of IGF-1 were decreased in both the PEE and PDE groups (*P* < 0.01; Fig. 3h). These results suggest that the fetal rats were exposed to higher GC levels that resulted in reduced IGF-1 levels.

#### Histological changes in fetal articular cartilage

In the control group, the cartilaginous matrix stained strongly with Safranin O (Fig. 3a,b), reflecting the high proteoglycan content. The chondrocytes in the articulating surface zone were tightly packed and had a typical flat, mesenchymal shape. The overall organization and structure of the joints in the PEE and PDE groups were not markedly altered; however, the number of chondrocytes in the articulating surface zone was decreased significantly (*P* < 0.05 for PEE, *P* < 0.01 for PDE; Fig. 3c), and defects in the articulating layer were observed, including hypocellular articular chondrocytes, gaps in the surface, and reduced Safranin O staining. The MOD of Safranin O staining of the articulating surface zone was significantly lower in the PEE (61.0% of control) and PDE (46.4% of control) groups compared to the control group (*P* < *0.01*; Fig. 3d). These results showed that consistent with PDE, PEE causes a reduction in chondrocytes and matrix synthesis in fetal articular cartilage.

#### IGF-1 pathway expression changes in fetal articular cartilage

**Figure 4:**
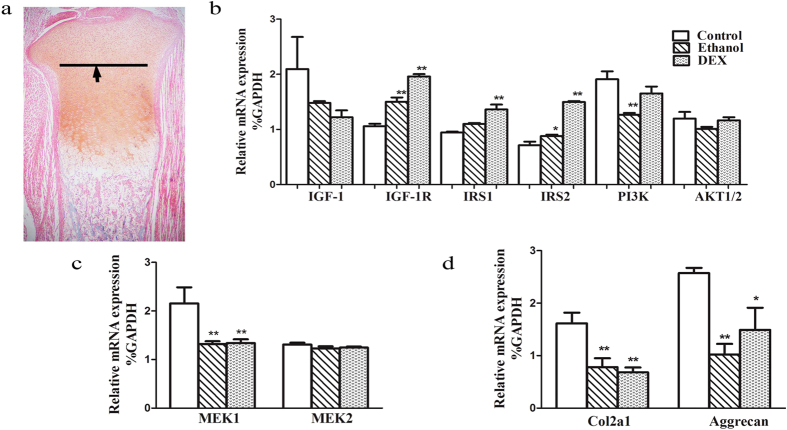
Effect of prenatal ethanol or dexamethasone (DEX) exposure on articular cartilage multiplex gene expression in female fetal rats. (**a**) The black arrows indicate the dividing line for cartilage removal in the experiment; (**b**) Insulin-like growth factor 1 (IGF-1) signaling pathway gene expression: IGF-1 receptor (IGF-1R), insulin receptor substrate 1/2 (IRS1/2), phosphatidylinositol 3-kinase (PI3K), and serine–threonine protein kinase (AKT1/2); (**c**) Proliferation-related gene expression: mitogen-activated protein kinase 1/2 (MEK1/2); (**d**) Extracellular matrix gene expression: collagen type II alpha 1 (Col2a1) and aggrecan. Mean ± S.E.M.; n = 8 litters from 8 pregnant rats. ^*^P < 0.05, ^**^P < 0.01 vs. control.

In the PEE group, the mRNA expression of IGF-1, PI3K and AKT exhibited a decreasing trend, while the mRNA expression of IGF-1 receptor (IGF-1R), insulin receptor substrate 1 (IRS1) and IRS2 showed an increasing trend. In the PDE group, the mRNA expression of IGF-1, PI3K, and AKT showed a decreasing trend, while the mRNA expression levels of IGF-1R, IRS1 and IRS2 were significantly increased compared to the control group (*P* < 0.05, *P* < 0.01). For cell proliferation genes (Fig. 4c), mitogen-activated protein kinase 1 (MEK1) mRNA expression levels were significantly lower in the PEE and PDE groups (*P* < 0.01), but the MEK2 mRNA expression level was not significantly different from that in the control group. The expression of aggrecan and Col2a1 mRNA was significantly lower in the PEE and PDE groups compared to the control group (*P* < 0.05, *P* < 0.01, Fig. 4d).

**Figure 5:**
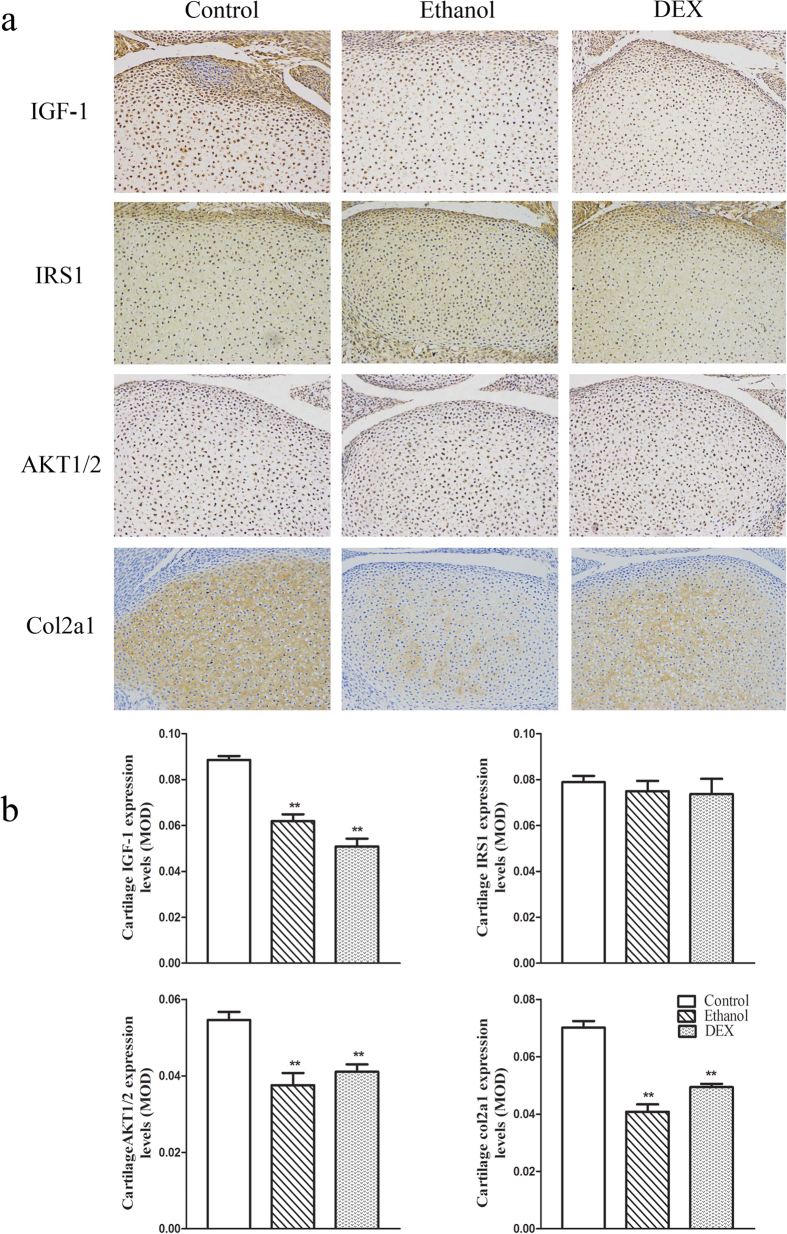
Effects of prenatal ethanol or dexamethasone (DEX) exposure on the protein expression of insulin-like growth factor 1 (IGF-1) signaling pathway components (insulin receptor substrate 1 (IRS1) and serine–threonine protein kinase 1/2 (AKT1/2)) and of collagen type II alpha 1 (Col2a1) in female fetal articular cartilage. (**a**) Representative immunohistochemistry (200×); (**b**) Mean optical density (MOD). Mean ± S.E.M.; n = 5 offspring from 8 pregnant rats. ^*^P < 0.05, ^**^P < 0.01 vs. control.

Immunohistochemistry analysis revealed that the protein expression of IGF-1, AKT1/2 and Col2a1 was significantly reduced to 69.9%, 68.7% and 58.2% of the control value, respectively, in fetuses in the PEE group (*P* < 0.01; Fig. 5a,b), and to 57.5%, 75.2% and 70.4%, respectively, in fetuses in the PDE group (*P* < 0.01; Fig. 5a,b). IRS1 protein expression showed no significant changes in the PEE and PDE groups.

## Discussion

Barker^19^ found that IUGR fetuses are prone to cardiovascular disease and type II diabetes through retrospective epidemiological surveys and proposed the theory of “the intrauterine origin of adult disease”. Accumulating evidence shows that MS (such as hypertension, hyperglycemia and dyslipidemia) has an intrauterine origin^20^. Currently, the etiology and pathogenesis of OA are indeterminate. Generally, OA has been reported to be related to age, mechanical friction, inflammation and matrix-degrading enzymes^21^. In addition, the incidence of OA is frequently linked to patient body weight and activity level, and OA usually occurs in the hip, knee and other weight-bearing joints. These factors interfere with epidemiological analyses, therefore masking the impact of birth weight on the incidence of OA. The current literature defines^22^ OA as a typical component of MS, and some reports^23^ even suggest that OA should not belong to the degenerative diseases but rather should belong to MS-associated diseases because OA has similar biochemical and inflammatory mechanisms and characteristics as MS. A variety of lipid and humoral factors associated with MS mediate the occurrence and development of OA. An increasing number of reports have identified positive correlations between OA and MS and between MS and IUGR; however, the mechanism by which IUGR increases the susceptibility to OA in adults has not been clearly elucidated.

We utilized a strenuous running exercise to induce OA. This method mimics the conventional pathogenesis of OA^24^ and avoids the use of man-made factors to destroy articular cartilage via chemical induction or surgery; therefore, it is an ideal method for inducing OA^24^. Our study showed that PEE significantly decreased fetal body weight, and IUGR offspring presented with postnatal catch-up growth. The gross morphology of the articular cartilage of the adult IUGR offspring showed no significant changes as assessed by India ink staining. However, after strenuous running, the rats showed obvious OA changes in the knees that were characterized by coarsened and increased lacunae on the articular cartilage surface, disordered cartilage cells and loss of extracellular matrix with a significantly increased Mankin score. Col2a1 is an important component of the extracellular matrix. Col2a1 is mainly secreted by chondrocytes and plays an important role in maintaining the physiological function of articular cartilage. The loss of Col2a1 is a major hallmark of articular cartilage degeneration^25^. In the adult animal experiments, the Col2a1 content exhibited a decreasing trend in non-running rats in the PEE group, but this decrease was more marked in running rats. These results suggest that PEE is harmful for cartilage functions in adult IUGR offspring and increases OA susceptibility.

Many studies have confirmed that low birth-weight/IUGR correlate with adult bone mass and osteoporotic fractures^26^,^27^. IUGR leads to delayed osteogenesis, adult peak bone density loss and other adverse consequences. Our previous studies confirmed that exposure to nicotine or caffeine during pregnancy resulted in the retardation of chondrogenesis^28^,^29^. However, no experimental studies have investigated the association between IUGR and adult OA susceptibility. Cartilage dysplasia may be a reason for the onset of OA^30^. Articular cartilage is mainly formed in the embryonic and neonatal periods^5^. More specifically, articular cartilage is a typical avascular, alymphatic and aneural tissue^31^. After maturation, little cell growth and proliferation occurs. Articular chondrocyte proliferation is visible only in exceptional cases (such as trauma, OA and acromegaly); however the structure and function of such chondrocytes is frequently abnormal^32^,^33^. After birth, the cell number within and the thickness of articular cartilage gradually decline with aging^34^. Therefore, the degree of intrauterine articular cartilage growth has an important influence on the quality of articular cartilage in adult offspring.

PEE can lead to developmental disorders in the fetal nervous system, heart, lungs and other organs, making the fetus susceptible to adult-associated diseases. The present study found that the number of knee joint articular chondrocytes was significantly decreased in IUGR fetuses with PEE. The cartilage matrix proteoglycan and Col2a1 content was significantly lower, suggesting fetal articular cartilage dysplasia. Based on the above theory, the incidence of OA among these individuals in adulthood may be higher. In our experimental rats, we confirmed the high susceptibility to OA, which is consistent with the above hypotheses. As a result, we propose that PEE increases OA susceptibility in adult offspring by inducing articular cartilage dysplasia.

IGF-1 phosphorylates PI3K/AKT to stimulate SOX9 expression, promote the synthesis of cartilage matrix Col2a1 and proteoglycan and maintain the characteristics of hyaline cartilage^35^. During development, systemic IGF-1 is primarily synthesized in the fetal liver and transported to other organs^36^. IGF-1 exerts paracrine and autocrine functions within cartilage during the second half of gestation^37^,^38^. GC down-regulates the IGF-1 signaling pathway and inhibits fetal chondrocyte proliferation^39^. Our previous studies demonstrated that fetal over-exposure to maternal CORT, which is induced by prenatal exposure to nicotine or caffeine, down-regulates IGF-1 signaling components in fetal growth plates and retards chondrogenesis^28^,^29^. Some studies have confirmed that the exposure to pharmacological doses of DEX inhibits chondrocyte proliferation, reduces matrix synthesis and thins the articular cartilage^40^. In addition, DEX can inhibit PI3K/AKT phosphorylation and protein expression in cartilage cells (cartilage cell line, ATDC5)^41^. Exogenous IGF-1 can reverse DEX-mediated cartilage cell apoptosis by activating the PI3K pathway^42^. These findings indicate that GC has a direct inhibitory function on the IGF-1 signaling pathway in chondrocytes, which leads to adverse outcomes.

To confirm that the fetal cartilage dysplasia caused by PEE is a result of over-exposure to GCs that inhibit the IGF-1 signaling pathway, a PDE model was used as a positive control. Our previous studies demonstrated that PDE inhibits the function of the maternal and fetal hypothalamic-pituitary-adrenal (HPA) axis through negative feedback^43^. Therefore, in the present study, although the blood CORT levels in the DEX group were low, the fetal rats were actually exposed to high levels of DEX (a synthetic GC). Consistent with the PDE group, PEE can decrease the serum and articular cartilage IGF-1 levels in fetal rats, and the expression of MEK1/2, PI3K/AKT and extracellular matrix proteins (Col2a1 and aggrecan) were decreased in fetal articular cartilage.

During intrauterine development, the plasticity and sensitivity of the fetus to the external environment is high. During the gradual adaptation to various environmental factors in utero, tissue and organ function and gene expression patterns result in the formation of a unique character and phenotypes that differ from the normal developmental pattern, and these persist throughout life. This process, with its persistent organizational effects, has been called “intrauterine programming”^44^. The lifelong programming of tissue or organ function and gene expression patterns during development may lead to numerous adverse effects in the adult^45^. In the present study, the protein expression of IGF-1, AKT1/2, SOX9 and Col2a1 in adult IUGR offspring with PEE remained lower in articular cartilage before and after strenuous running, indicating that the local IGF-1 signaling pathway in articular cartilage was low-functioning, consistent with the changes that occurred during the fetal period. These results suggest that the local articular cartilage IGF-1 signaling pathway may be programmed to be low-functioning in fetuses with PEE-induced IUGR. This expression state weakened the articular cartilage resistance to external stress in adults, eventually leading to OA susceptibility.

## Conclusion

The present study demonstrates that PEE resulted in IUGR, articular cartilage dysplasia and increased susceptibility to adult OA in the offspring. The underlying mechanisms may be associated with the elevated exposure to maternal GC and a low-functioning local IGF-1 pathway. Overall, these data bring new insights into novel intrauterine phenomena of adult OA and its interrelationship with the IUGR.

## Materials and Methods

### Chemicals and reagents

Ethanol (analytical pure grade) was obtained from Zhen Xin Co. Ltd. (Shanghai, China). Isoflurane was purchased from Baxter Healthcare Co. (Deerfield, IL, USA). Rat CORT ELISA kits were obtained from Assay pro LLC. (Saint Charles, MO, USA). Rat IGF-1 ELISA kits were purchased from RD Systems, Inc. (Minneapolis, MN, USA). TRIzol reagent kits were obtained from Omega Bio-Tek (Doraville, USA). GeXP multiplex gene expression analysis kits were purchased from Beckman-Coulter Inc. (Fullerton, CA, USA). The oligonucleotide primers for rat Q-PCR (PAGE purification) and GeXP multiplex gene expression analysis (HPLC purification) were synthesized by Sangon Biotech Co., Ltd. (Shanghai, China). Polyclonal rat IGF-1, IRS1, AKT1/2, SOX9 and Col2a1 antibodies were purchased from Santa Cruz Biotechnology, Inc. (Santa Cruz, CA, USA). Other chemicals and agents were of analytical grade.

### Animals and treatment

Specific pathogen-free (SPF) Wistar rats (females: 180 ~ 220 g; males: 260 ~ 300 g) were obtained from the Experimental Center of Hubei Medical Scientific Academy (No. 2010–2012, Hubei, China). This experiment was performed in the Center for Animal Experimentation of Wuhan University (Wuhan, China), which has been accredited by the Association for Assessment and Accreditation of Laboratory Animal Care International (AAALAC International). All the animal experimental procedures were approved by the Chinese Animal Welfare Committee and were performed in accordance with their Guidelines for the Care and Use of Laboratory Animals.

Animals were housed (room temperature: 18–22 °C; humidity: 40–60%), acclimated, and mated. Mating was confirmed by the appearance of sperm in a vaginal smear, and this day was recorded as gestational day (GD) 0. Pregnant rats were transferred to individual cages and randomly divided into the control, PEE and PDE groups. From GD11 to GD20, the PEE group was treated with 4 g/kg.d ethanol by oral gavage^46^, the PDE group was treated with 1 mg/kg.d DEX subcutaneously^47^, and the control group received the same volume of distilled water by oral gavage.

For experiments on fetal rats, 8 pregnant rats with 10–14 live fetuses were randomly selected from each group, anesthetized with isoflurane, and euthanized on GD20. The female fetuses were quickly removed and weighed. IUGR was diagnosed as previously described^48^. Fetal blood samples and knee joints were collected as in our previous studies^28^. Briefly, fetal blood samples were collected, serum was isolated, and fetal knee joints were separated under a dissecting microscope and collected. The samples collected from littermates were pooled, immediately frozen in liquid nitrogen, and stored at −80 °C for subsequent analyses. Fetal left knee joints were fixed in 4% paraformaldehyde until further analysis.

For experiments on adult rats, some pregnant rats in the control and PEE groups were allowed to deliver normally (GD21). On postnatal day 1 (PD1), IUGR was diagnosed, and the number of pups was normalized to 8 pups per litter (each group retained 8 litters) to assure adequate and standardized nutrition until weaning (PW4). After weaning, two female pups per litter were randomly selected from each group (each group contained 16 rats for the experiments). These offspring were weighed weekly until PW18, and the corresponding growth rates were calculated as follows: Gain rate of body weight (%) = [(body weight at PWX-body weight at PW1)]/body weight at PW1 × 100^49^. At PW17, each group was randomly divided into two subgroups: a running group (30 km, n = 8) and a non-running group (n = 8). For the running group, the rats were acclimated to the treadmill by gradually increasing the running speed and time for 7 days on a rodent treadmill machine (Huaibei Zhenghua Company, Anhui, China). On day 8 and thereafter, rats were forced to run for 55 min a day at 20 m/min, with a 12 m/min warm-up for the first 10 min; the rats ran 30 km in 6 weeks^24^. At PW24, all the experimental animals were anesthetized with isoflurane and euthanized. The left femoral condyles and tibial plateau were carefully dissected and stained separately with India ink to identify the location, size and severity of articular cartilage destruction^50^. The right knee joints were fixed with 4% formaldehyde for histological and immunohistochemical assays.

### ELISA assay for blood CORT and IGF-1 concentrations

Fetal serum CORT and IGF-1 concentrations were determined using ELISA assay kits according to the manufacturer’s instructions^16^. The intra- and inter-assay coefficients of variation were 5.0% and 7.2% for CORT and 4.3% and 6.0% for IGF-1.

### Histological and immunohistochemical assays

Fetal rat knee specimens were fixed in 4% paraformaldehyde at pH 7.4 for 3 days and then processed for paraffin embedding. Adult knee joints were fixed in 4% paraformaldehyde, decalcified in a 20% ethylenediamine tetraacetic acid solution for 21 days, and then embedded in paraffin wax. The knee joints were sectioned sagittally at a thickness of 5 μm. Safranin O/fast green staining and immunohistochemical assays were performed by standard procedures^28^. The sections were visualized and photographed with an Olympus AH-2 light microscope (Olympus, Tokyo, Japan). Five Safranin O-stained sections of the fetal tibia were randomly chosen to measure the proteoglycan levels in each group. Each Safranin O-stained adult femur and tibias section was evaluated using the Mankin histological grading system, which includes structure, cell number, cartilage matrix staining and tide mark (score: range from 0 to 14)^51^. Immunohistochemistry was performed with a DAB staining kit (GeneTech Company, Ltd., Shanghai, China) to determine the protein expression of IGF-1, IRS-1, AKT1/2, SOX9 and Col2a1 in the fetal and adult knee joints. Rabbit anti-rat polyclonal primary antibodies and goat anti-rabbit IgG secondary antibodies were used. All of the images were captured and analyzed using Image Pro Plus software (version 6.1, Media Cybernetics, Silver Spring, USA). The staining intensity was determined based on the MOD in 10 different fields for each sample.

### Total RNA extraction and multiplex gene expression analysis

Detailed protocols for total RNA extraction were reported in our previous study^28^. A multiplex analysis containing 3 housekeeping genes (GAPDH, β-actin and tubulin) and 10 target genes (IGF-1, IGF-1R, IRS1, IRS2, PI3K, AKT1/2, MEK1, MEK2, Aggrecan and Col2a1) was performed on the GenomeLab GeXP Genetic Analysis System (Beckman-Coulter) using GenomeLab^TM^ eXpress Profiler software (Beckman-Coulter, Fullerton, CA)^52^. Multiplex optimizations (e.g., primer validation and attenuation) were completed according to the manufacturer’s instructions as previously reported by Shen, *et al.*^16^. Briefly, a primer pair was considered valid if only one PCR product differed from its predicted size by less than one nucleotide. The list of genes and primer pairs are provided in Table 1. To account for the different scale of expression of each gene, the proportion of each reverse primer in the multiplex reverse transcriptional reaction was adjusted to obtain similar peak signals for each gene. Reverse transcription was performed using 100 ng of RNA as a template. Real-time PCR amplification was performed using a mix of the forward primers, and the resulting reactions were analyzed by capillary electrophoresis on the GenomeLab GeXP using the GeXP Start kit reagents. Relative RNA expressional levels were calculated relative to a pooled RNA standard using the GeXP Quant Tool software, and the expression levels were normalized to those of the housekeeping genes.

### Statistical analysis

SPSS 17 (SPSS Science Inc., Chicago, Illinois) was used for data analysis. Quantitative data are expressed as the mean ± S.E.M. The longitudinal changes in body weight and growth rate were analyzed by two-way ANOVA with repeated measures. The Mankin scores of OA were compiled for each joint and subjected to a Mann–Whitney *U*-test. The immunohistochemistry data on the adult rats were analyzed by a 2 × 2 factorial analysis. Enumeration data, such as the IUGR rate, were statistically analyzed after arcsine square root transformation^28^. The comparisons among fetal groups were performed using one-way ANOVA followed by Dunnett’s post hoc *t*-tests. *P* < 0.05 was considered to be statistically significant.

## Additional Information

**How to cite this article**: Ni, Q. *et al.* Prenatal ethanol exposure increases osteoarthritis susceptibility in female rat offspring by programming a low-functioning IGF-1 signaling pathway. *Sci. Rep.* **5**, 14711; doi: 10.1038/srep14711 (2015).

## Figures and Tables

**Table 1:** Primer sequences of GeXP analysis genes.

Genes	Forward primers	Reverse primers
IGF-1	AGGTGACACTATAGAATACCAGCTGTTTCCTGTCTACAG	GTACGACTCACTATAGGGAAGATGTGAAGACGACATGATGT
IGF-1R	AGGTGACACTATAGAATACAAGACAGAAGTCTGCGGTG	GTACGACTCACTATAGGGACCGGGTCTGTGATATTGTAGG
IRS1	AGGTGACACTATAGAATAGGACCGTCAATAGCTTAACTGG	GTACGACTCACTATAGGGAGTCACAGTGCTTTCTTGTTGCT
IRS2	AGGTGACACTATAGAATATGTCCCATCACTTGAAAGAAG	GTACGACTCACTATAGGGACCTGCCTCTTGGTTCCTTATC
MEK1	AGGTGACACTATAGAATACAAGATGCCCAAGAAGAAGC	GTACGACTCACTATAGGGAAGCCATAACCAGGCCAGAT
MEK2	AGGTGACACTATAGAATATCATTAAGAACCCAGCAGAGC	GTACGACTCACTATAGGGATCTCCACTTTGTTTGCTTTTCC
PI3K	AGGTGACACTATAGAATACGATGGAATTGGAACGAGTG	GTACGACTCACTATAGGGACCAGACTTTCAAGTCGTGCA
AKT1/2	AGGTGACACTATAGAATAATGAACGACGTAGCCATTGTG	GTACGACTCACTATAGGGAATGATGAAGGTGTTGGGCCT
Aggrecan	AGGTGACACTATAGAATACGGACTGAAGTTCTTGGAGG	GTACGACTCACTATAGGGAGGTTGAGGGATGCTCACACT
Col2a1	AGGTGACACTATAGAATATCAAGGAGAAGCTGGACAGAA	GTACGACTCACTATAGGGACCCAGGGTTGCCATTAGAAC
GAPDH	AGGTGACACTATAGAATATCTCTGCTCCTCCCTGTTCTAG	GTACGACTCACTATAGGGAGGTCAATGAAGGGGTCGTTG
β-actin	AGGTGACACTATAGAATAGTCCACCCGCGAGTACAAC	GTACGACTCACTATAGGGACCCACGTAGGAGTCCTTCTG
Tublin	AGGTGACACTATAGAATATGTAAGAAGCAACACCTCCTC	GTACGACTCACTATAGGGACAGTGCGAACTTCATCAATAAC

IGF-1, insulin-like growth factor 1; IGF-1R, insulin-like growth factor-1 receptor; IRS1/2, insulin receptor substrate 1/2; MEK1/2, mitogen-activated protein kinase 1/2; PI3K, phosphatidylinositol 3-kinase; AKT1/2, serine–threonine protein kinase; Col2a1, collagen type II alpha 1; GAPDH, glyceraldehyde 3-phosphate dehydrogenase.
